# Tolerated Re-Challenge of Immunotherapy in a Patient with ICI Associated Myocarditis: A Case Report and Literature Review

**DOI:** 10.3390/medicina59111946

**Published:** 2023-11-03

**Authors:** Walid Shalata, Zoé Gabrielle Attal, Rajeh Shhadi, Amjad Abu Salman, Ashraf Abu Jama, Sondos Shalata, Kais Halumi, Alexander Yakobson

**Affiliations:** 1 The Legacy Heritage Cancer Center & Larry Norton Institute, Soroka Medical Center, Beer Sheva 84105, Israel; 2 Faculty of Health Sciences, Ben-Gurion University of the Negev, Beer Sheva 84105, Israel; 3 Medical School for International Health, Ben Gurion University of the Negev, Beer Sheva 84105, Israel; 4 Cardiology Division, Soroka Medical Center, Beer Sheva 84105, Israel; 5 Nutrition Unit, Galilee Medical Center, Nahariya 22000, Israel

**Keywords:** myocarditis, cardiac toxicity, inhibitors, immune checkpoint inhibitors (ICIs), immune-related adverse events (IRAE), cytotoxic T-lymphocyte-associated protein 4 (CTLA-4), programmed cell death protein 1 (PD-1), programmed death-ligand 1 (PD-L1)

## Abstract

Many different types of cancer can be treated with immunotherapy drugs called immune checkpoint inhibitors (ICIs). These drugs have altered the landscape of cancer treatment options since they function by triggering a stronger immune response to malignancy. As expected, ICIs’ modification of immune regulatory controls leads to a wide range of organ/gland-specific immune-related side effects. These adverse effects are uncommonly deadly and typically improve by discontinuing treatment or administering corticosteroid drugs. As a result of a number of factors—including a lack of specificity in the clinical presentation, the possibility of overlap with other cardiovascular and general medical illnesses, difficulties in diagnosis, and a general lack of awareness—the true incidence of ICI-associated myocarditis is likely underestimated. Currently, protocols for the surveillance, diagnosis, or treatment of this condition are unclear. Several questions remain unanswered, such as how to best screen for this rare toxin, what tests should be run on patients who are suspected of having it, how to treat myocarditis once it has developed, and who is at most risk. In this article, we provide a case study of ICI-associated myocarditis and explain its key characteristics and treatment options.

## 1. Introduction

A melanoma is a skin tumor described as the deadliest type of skin cancer. It is generally treated by surgical resection, immunotherapy, chemotherapy, and other therapeutic approaches [1,2,3]. In metastatic melanoma, immunotherapy is preferred and is divided into four categories. These include biological medications, therapeutic vaccinations immunomodulation, adoptive cell therapy, and immune check point inhibitors [2,3,4,5]. The latter group have become the backbone of treatment in recent years specifically with the development of anti-programmed cell death 1 (PD-1) and anticytotoxic T lymphocyte associated protein 4 (CTLA-4) [3,4,5,6]. In advanced melanoma, a combined approach using ipilimumab and nivolumab has been found to have higher survival rates compared to ipilimumab monotherapy (median survival 11.5 vs. 2.9 months) [7]. Additionally, recent clinical trials have demonstrated that tebentafusp shows a survival benefit in the treatment of metastatic uveal melanoma. The overall survival rate with tebentafusp stood at 73% compared to 59% for the control group (treated with either pembrolizumab, dacarbazine, or ipilimumab) [8].

Immune checkpoint inhibitors (ICIs) have been associated with the development of immune-related adverse events (irAEs) because of the heightened immune activity they induce. The gastrointestinal tract, endocrine glands, skin, and liver are the most commonly affected organs, but any organ system may be vulnerable. As such, a large scale safety meta-analysis demonstrated that ICI therapy typically increases the risk of dyslipidemia, pericardial diseases, MI, cerebral arterial ischemia, and myocarditis in 3–20 per 1000 patients [9]. There have been recent reports of cardiotoxicity in the form of myocarditis [7,8,9,10,11,12,13,14,15,16,17,18,19]. However, the extent to which ICIs contribute to the development of myocarditis is not well understood. According to pharmacovigilance data, 0.27% of patients on combination therapy and 0.9% of patients on a single ICI developed myocarditis. Nevertheless, a recent retrospective case–control study found that 1% of individuals treated with ICIs developed myocarditis. The risk factors that increase the likelihood of developing myocarditis due to ICIs are not well-established. The presence of an underlying autoimmune illness, as well as heart disease and diabetes, may increase the risk of myocarditis associated with ICI therapy. Furthermore, myocarditis has been observed to be more frequently present in combination therapy and with anti-PD-1/anti-CTLA-4 therapy. The incidence of myocarditis varies between different classes of ICIs, with anti-PD-1 agents having the lowest incidence (0.5%) and anti-CTLA-4 monotherapy having the highest (3.3%). However, it is important to note that these differences may be overestimated and that both anti-PD-1 and anti-PD-L1 agents have a similar toxicity profile [6,10,11,12,13,14,15,16,17,18,19].

It is generally acknowledged that ICI-associated myocarditis tends to manifest early, with a median onset of 1–2 months and most cases occurring within 3 months of initiating ICI therapy. This suggests the presence of a pre-existing subclinical immunological risk factor. Although cardiovascular adverse events tend to occur early in treatment, they can occur at any time. Symptoms may be mild and nonspecific, such as fatigue, myalgia, chest pain, and shortness of breath, or more severe, such as syncope and sudden cardiac death. Myocarditis may also manifest as tachyarrhythmias (atrial or ventricular) or cardiac block. Subclinical or smoldering myocarditis with minimal symptoms may also occur, although reports of fulminant myocarditis with heart failure and arrhythmias are more common [6,10,11,12].

In this review article, we describe a case of ICI related grade 3 myocarditis in a patient suffering from metastatic melanoma. Our patient had complete recovery from the myocarditis after cessation of immunotherapy and introduction of corticosteroids. However, a rechallenge with ICI was faced in 2021 when the patient had recurrence of her melanoma and no myocarditis was observed.

## 2. Case Presentation

The patient had a history of hypertension and type 2 diabetes mellitus and had previously undergone a total thyroidectomy in 2016 for papillary carcinoma of the thyroid and excision of a benign endometrial polyp in 2004; furthermore she had no family history of cancer and history of smoking.

**Figure 1:**
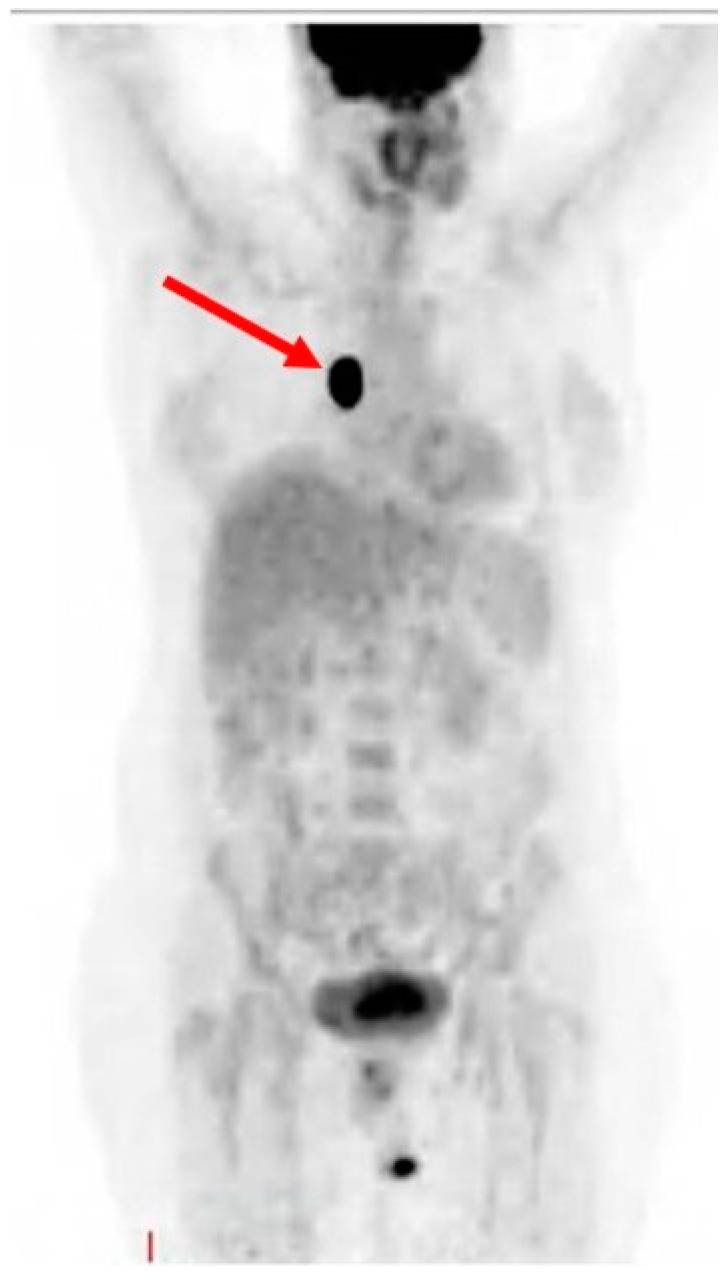
PET–CT scan showing hypermetabolic absorption in the vertebrae area (red arrow).

On 19 December 2017, a biopsy of the nevus revealed metastatic malignant melanoma and a subsequent total body PET–CT scan revealed hyper metabolic absorption in the vertebral bodies and soft tissue at levels D5, D7, and D9 (the area of the nevus), consistent with malignant disease (Figure 1).

In response to the diagnosis, the patient was initiated on systemic immunotherapy with nivolumab 200 mg, administered intravenously every 2 weeks. Following the fourth cycle of treatment, the patient developed itching and a mild skin rash. However, one week later, the patient was admitted to the Department of Internal Medicine because of the onset of headache, weakness, and an increase in liver enzymes with GOT (AST) 53 U/L (normal 0–31) and GPT (ALT) 56 U/L (normal 0–34). Further evaluation, including chest radiography, abdominal ultrasound, and CT of the abdomen revealed no evidence of pathological findings; in addition, CBC and chemistry blood tests showed no extra pathological findings except for the previously noted elevated liver enzymes.

The patient was discharged from hospital after 10 days and a repeat PET–CT test performed one week later showed stable disease.

Given the patient’s response to treatment, it was decided to add ipilimumab 75 mg and administer it in combination with the nivolumab 200 mg every 3 weeks.

**Figure 2:**
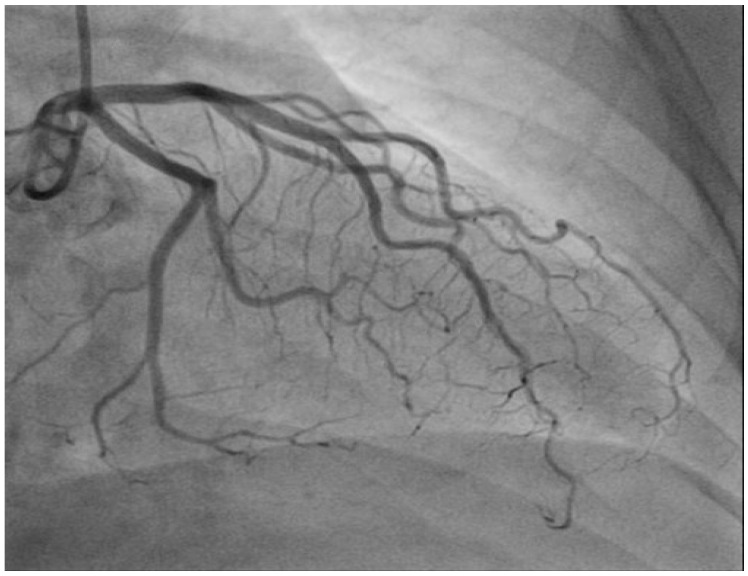
Electrocardiography, angiogram of the coronary arteries showing no pathological findings.

In May 2018, the patient received the second cycle of nivolumab plus ipilimumab. However, two weeks later, the patient was admitted to the Department of Internal Medicine with chest pain, fever (38.7 °C), and dyspnea. Further evaluation, including electrocardiography and an angiogram of the coronary arteries, revealed no evidence of pathological findings (Figure 2). An echocardiogram showed normal LV systolic function with an ejection fraction of 65% and signs of impaired LV relaxation and mild mitral regurgitation, with no additional pathological findings.

The patient presented with an elevated troponin level, initially measuring 326 ng/L and subsequently decreasing to 256 ng/L. Steroid therapy was initiated (prednisone 1.5 mg/kg).

A ventilation/perfusion lung scan was conducted and revealed no abnormalities.

A complete blood count and blood chemistry panel were also conducted, yielding normal results. However, a mild elevation in liver enzymes (GOT [AST] 80 U/L, GPT [ALT] 73 U/L), lactate dehydrogenase (843 U/L), absolute eosinophilia of 5.34 × 10^3^/uL (normal 0–0.8), and eosinophilia of 38.8% were noted.

Virology testing was performed, revealing negative results for DNA PCR for EBV and CMV, as well as HIV. Additional investigations, including an antinuclear antibody test, anti-neutrophil cytoplasmic antibody test, and an assessment of immunoglobulin levels, were conducted and also revealed no abnormalities. A blood culture was conducted, yielding no growth of bacteria.

**Figure 3:**
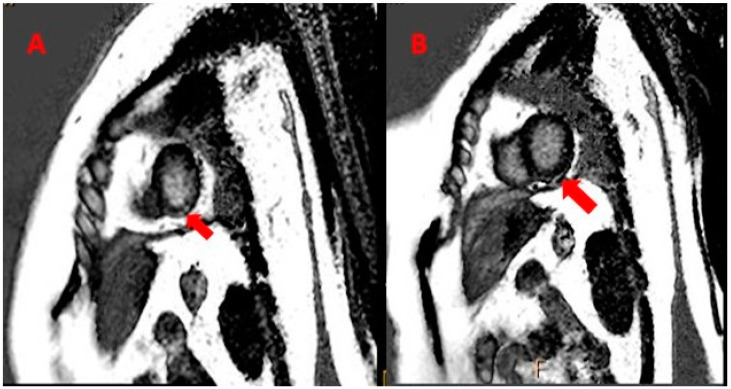
MRI of the heart, showing LGE in inferior lateral left ventricular (red arrows) (**A**). In addition, minimal pericardial effusion was noted (red arrows) (**B**).

A CT scan of the spine and MRI of the heart were performed, revealing Sub-endocardial Late gadolinium Enhancement (LGE) in the inferior lateral left ventricular (Figure 3A); mild mitral regurgitation and minimal pericardial effusion were noted (Figure 3B). After consultation with a cardiologist, a diagnosis of myocarditis (stage 3) was made on clinical grounds.

On August 2018, a PET–CT scan was performed, revealing weak hyper metabolic absorption in soft tissue in the area of the nevus. A multidisciplinary team excised the nevus and continuously monitored the patient.

In April 2021, several nevi were observed on the patient’s upper and middle back, and a biopsy revealed metastatic melanoma. A multidisciplinary conference, comprising an oncologist, cardiologist, and immunologist, was conducted to determine the appropriate course of treatment considering her history with irAEs (grade 3 myocarditis). It was decided to re-initiate therapy with nivolumab alone in order to minimize the risk of immune-related adverse events.

The treatment was re-initiated on May 2021, and since then the patient has been under regular follow-up with a cardiologist and immunologist (monthly) and an oncologist (every 2 weeks), including cardiac MRI and ECO (every 2–3 months), as well as blood tests for troponin and proBnp (monthly).

**Figure 4:**
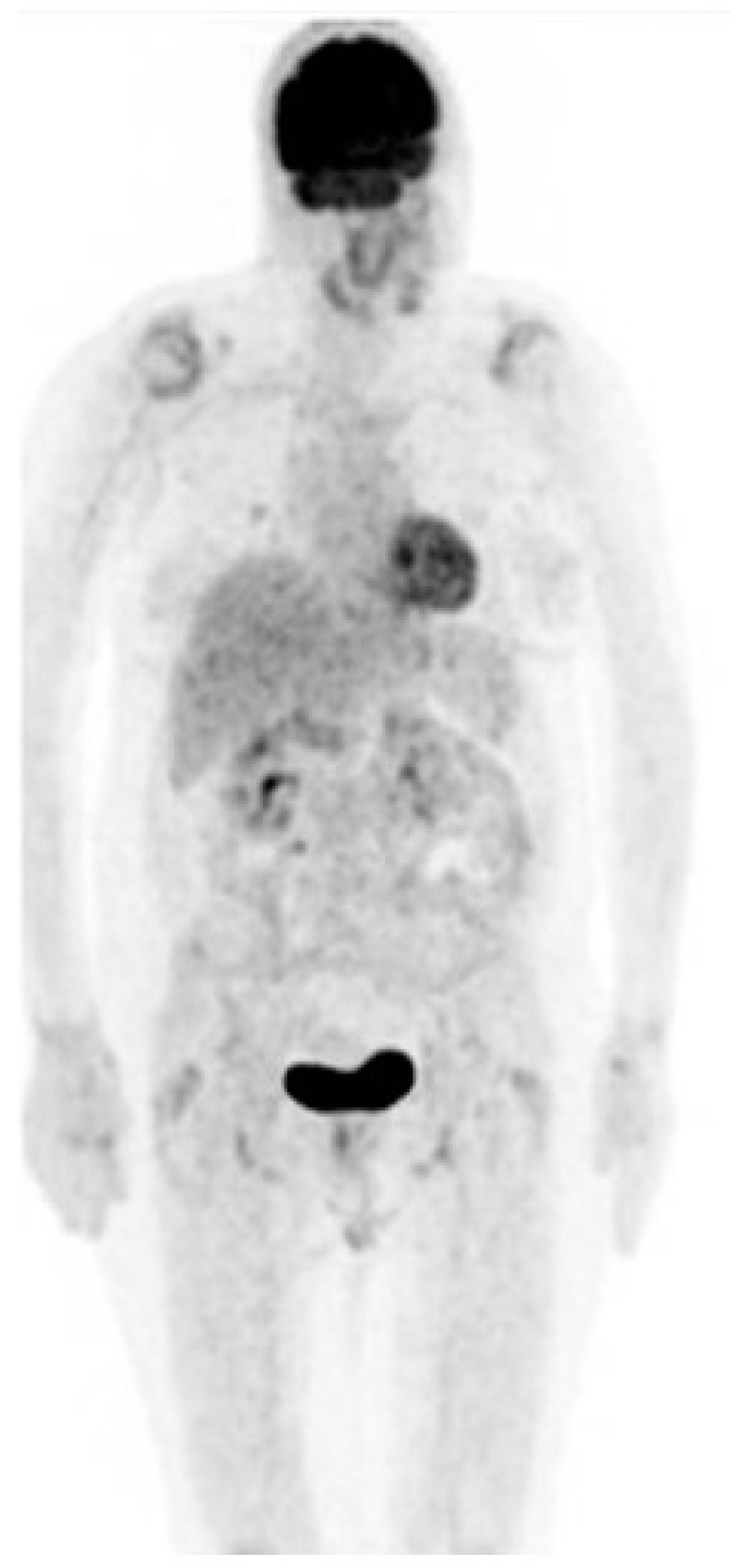
PET–CT scan showing no evidence of disease.

As of August 2023, the patient had received 41 cycles of therapy without adverse effects incidents and a recent PET–CT scan showed a complete response to treatment (Figure 4).

## 3. Diagnosis

There are currently no established protocols for the diagnosis and management of this relatively novel condition; however, as more is learned, therapeutic strategies will likely change to reflect this.

The patient’s electrocardiogram (ECG) and troponin levels must be tested quickly if they exhibit symptoms consistent with myocarditis. Although these tests are commonly used to diagnose myocarditis, it is crucial to note that they do not have the sensitivity and specificity required for diagnosis and can be abnormal because of other cardiovascular disorders. Sinus tachycardia, QRS/QT prolongation, conduction abnormalities, diffuse T-wave inversion, Q waves, ventricular arrhythmias, and local or diffuse ST elevation are all examples of nonspecific ECG symptoms that may be present in a patient with myocarditis. A normal electrocardiogram (ECG) does not rule out myocarditis, despite the fact that abnormalities in the ECG are present upon presentation in the vast majority of individuals with myocarditis. Similar to how an increased troponin is not specific for myocarditis, a normal troponin does not rule out ICI-related myocarditis, even though it is associated with most reported fulminant cases. However, elevated troponin levels have been found to be prognostic, with greater troponin levels related to worse cardiovascular outcomes. This extends the usefulness of troponin levels beyond the diagnostic realm and into the realm of patient management. Patients with symptoms should have other cardiac biomarkers measured, such as BNP or NTproBNP, which are indicators of myocardial strain but may be normal in certain phenotypes [6,13,14,15,16,17,18,19,20,21,22,23,24,25,26,27,28,29,30,31,32,33,34,35,36,37,38,39,40,41,42,43,44,45,46,47].

Because of its widespread availability and ease of performance, an echocardiography is a routine first-line test for the evaluation of patients with suspected ICI-associated myocarditis. However, even with fulminant myocarditis, the LVEF can be normal, and this does not rule out the possibility of a significant adverse cardiac event. Coronary ischemia must be ruled out in all patients presenting with new cardiovascular symptoms, an abnormal ECG, and an increased cTn. This can be done using invasive coronary angiography, a cardiac CT, or, less ideally, a stress test, depending on the clinical presentation. In order to rule out other potential causes of myocarditis, a viral serology panel, which includes influenza testing, should be undertaken. Myocarditis can best be diagnosed using a CMR, the most reliable noninvasive diagnostic. CMRs advantages include their high spatial resolution and their supplemental capacity for tissue characterization. Myocarditis is detectable on CMRs’ T1- and T2-weighted images because of the increased water content and cellular necrosis inflamed hearts have due to increased capillary permeability. Our case was supported by the detection of late gadolinium enhancement. These CMR criteria, when used together, have a sensitivity of 76% and a specificity of 96% for diagnosing myocarditis. Magnetic resonance imaging (CMR) has also been proven to be useful in general cases of myocarditis for risk stratification and prognostication. However, CMR is not routinely used for every patient with suspected myocarditis because of its limited availability and the difficulties in performing this very lengthy test in severely unwell patients. Myocarditis cannot be ruled out even if echocardiography or CMR results are normal [6,23,24,25,26,27,28,29,30,31,32,33,34,35,36,37,38,39,40,41,42,43,44,45,46,47].

Myocarditis is best diagnosed by a surgical endomyocardial biopsy. Despite being the ‘gold standard’, this test is not performed routinely because of the invasive nature of the procedure, the danger of heart perforation, and the limited scope of the biopsy sample. Histological analysis of a biopsy from the afflicted area may reveal inflammatory infiltrates (often T-cell-predominant lymphocytic infiltrate) in the myocardium, which are not consistent with ischemia damage from coronary artery disease. In order to increase the test’s sensitivity, immunostains for cell-specific markers including T lymphocytes (CD3), macrophages (CD68), or human leukocyte antigens may be used [6,35,36,37,38,39,40,41,42,43,44,45,46,47,48].

Patients who are suspected of having or who have been diagnosed with ICI-associated myocarditis should be continuously monitored using cardiac telemetry and ECGs because of the risk of tachy- and bradyarrhythmias [6,13,14,15,16,17,18,19,20,21,22,23,24,25,26,27,28,29,30,31,32,33,34,35,36,37,38,39,40,41,42,43,44,45,46,47,48].

In a patient who is thought to have myocarditis, it is crucial to think about a wide range of possible causes. Since pneumonia is another irAE that can present with similar symptoms, it is important to rule it out during the diagnostic process, especially if the workup for myocarditis turns up negative. There is a good chance that many of these patients have previously been exposed to various medicines with the potential to be cardiotoxic, all of which may have contributed to the development of cardiac dysfunction. Combination therapy with BRAF/MEK inhibitors, which is linked to LV systolic dysfunction, may have been given to patients with BRAFV600 mutant metastatic melanoma. It is also important to evaluate the possibility of alternative diagnoses. For instance, heart block and heart failure have been documented in patients on immunotherapy for sarcoidosis. This condition can have comparable effects on the cardiovascular system as ICI-associated myocarditis [6,25,26,27,28,29,30,31,32,33,34,35,36,37,38,39,40,41,42,43,44,45,46,47,48].

## 4. Treatment

The management of cardiac immune-related adverse events (irAEs) associated with immune checkpoint inhibitors (ICIs) remains a complex and evolving field. While there are currently no established protocols for the diagnosis and management of this relatively novel condition, therapeutic strategies will likely change to reflect the increasing knowledge in this area. The foundation of treatment for ICI-associated myocarditis involves the cessation of ICI therapy and initiation of immunosuppression. Given the possibility for rapid progression to fulminant disease with cardiovascular compromise, it is suggested that immunosuppressive therapy be initiated without waiting for confirmation testing because of the importance of timing of treatment [6,17,18,19,20,21,22,23,24,25,26,27,28,29,30,31,32,33,34,35,36,37,38,39,40,41].

When treating an acute phase patient, high-dose corticosteroids are typically administered as the first line of treatment (e.g., methylprednisolone 1000 mg daily for 3 days, then prednisone 1 mg/kg), with the aim of improving left ventricular systolic function and reducing the risk of severe adverse cardiac events [6,17,18,19,20,21,22,23,24,25,26,27,28,29,30,31,32,33,34,35,36,37,38,39,40,41,42,43,44,45,46,47,48].

However, if high-dose steroids fail to alleviate myocarditis symptoms, other options such as infliximab, anti-thymocyte globulin, intravenous immunoglobulin, and plasma exchange may be considered [6,18,19,20,21,22,23,24,25,26,27,28,29,30,31,32,33,34,35,36,37,38,39,40,41].

It is essential to acknowledge that the available data on the efficacy of infliximab is inconclusive, and its use has been associated with an increased risk of heart failure among individuals with rheumatoid arthritis. In cases where the patient’s condition deteriorates significantly, alternative treatments such as anti-thymocyte globulin, intravenous immunoglobulin, and plasma exchange may be considered. In stable patients, incorporating supplementary therapy with immunosuppressive agents such as tacrolimus or mycophenolate mofetil, which have been demonstrated to be effective in preventing cardiac allograft rejection, should be considered in cases of high-grade myocarditis on biopsy or lack of response to corticosteroid therapy. If the left ventricular ejection fraction (LVEF) is low, it is also crucial to initiate treatment for heart failure and arrhythmias in a conventional manner. While the optimal duration of treatment is yet to be determined, it is reasonable to continue immunosuppressive therapy until symptom resolution and normalization of LVEF, biomarkers, and conduction abnormalities [6,19,20,21,22,23,24,25,26,27,29,30,31,32,33,34,35,36,37,38,39,40,41,42,43,44,45,46,47,48,49,50,51,52,53,54].

The emergence of cardiovascular adverse events (irAEs) in cancer patients receiving immune checkpoint inhibitors (ICIs) poses a significant challenge because of its potential impact on cancer treatment and prognosis. Cardiac complications can lead to serious repercussions or even death, while interruptions in cancer therapy can increase the risk of disease progression. Retrospective studies have shown that patients with advanced melanoma or non-small cell lung cancer who initially responded positively to ICIs but had to discontinue treatment because of irAEs may not need to restart ICI therapy [30,31,32,33,34,35,36,37,38,39,40,41,42,43,44,45,46,47]. The decision to rechallenge with ICI therapy following the development of ICI-associated myocarditis is complex and personalized decisions should be made through multidisciplinary discussions, taking into account the patient’s cancer status, response to immunotherapy, availability of alternative effective therapy, severity of cardiotoxicity, and regression of toxic effects. Guidelines recommend discontinuing immunotherapy in cases of life-threatening (grade 4) and severe (grade 3) adverse events. If rechallenge with immunotherapy is deemed necessary, monotherapy with a different drug and close cardiovascular monitoring should be considered. According to a retrospective study of limited registry data, monotherapy with an anti-PD1 drug has the lowest risk of cardiotoxicity. Similar results were found in a retrospective investigation of patients with melanoma, which demonstrated that anti-PD1 medication can be safely administered following the occurrence of severe side events associated with ipilimumab (anti-CTLA4) or combination therapy including CTLA4/PD1 [13,14,15,16,17,18,19,20,21,22,23,24,25,26,27,28,29,30,31,32,33,34,35,36,37,38,39,40,41,42,43,44,45,46,47,48,49,50,51,52,53,54].

## 5. Screening and Surveillance

When there is a high risk of cardiotoxicity from cancer treatments, it is essential to implement both screening and surveillance measures. Specifically, an assessment of left ventricular ejection fraction (LVEF) prior to initiating anthracycline therapy is recommended. However, measuring LVEF before starting immune checkpoint inhibitor (ICI) therapy may not be useful, as data suggests that 70% of individuals who developed myocarditis while receiving ICI therapy had normal LVEF levels before treatment. The majority of studies report abnormal electrocardiograms (ECGs) and cardiac troponin (cTn) at presentation. Therefore, serial ECG and cTn monitoring as a form of surveillance may be considered [6,36,37,38,39,40,41,42,43,44,45]. Because of the short median time to myocarditis, measuring cTn levels at the beginning and end of each cycle may be beneficial. If an increased cTn level suggests myocarditis, consultation with cardiology/cardio-oncology should be sought immediately for further assessment. Other considerations include identifying the patient population that should be monitored, and for how long. Patients receiving adjuvant or neoadjuvant therapy, those using combination ICI regimens, and those receiving ICIs in conjunction with other treatments known to have cardiovascular toxicities may all benefit from surveillance [6,23,24,25,26,27,28,29,30,31,32,33,34,35,36,37,38,48,49,50,51,52,53,54].

## 6. Discussion

### 6.1. Pathophysiology

The precise mechanism of immune-related adverse events (irAEs) remains unclear. However, the idea that a shared antigen may play a role is supported by emerging evidence that high-frequency T-cell receptor sequences are shared by both tumors and heart muscle. Additionally, the relatively quick onset of myocarditis following the initiation of ICI therapy, as well as the involvement of a small number of patients without a clear cause, lends support to theories about the contribution of pre-existing illnesses to the development of myocarditis. Studies in genetic models have shed some light on the role of CTLA-4 and PD-1 in protecting the heart from immune-mediated injury after stress. Specifically, the deletion of PD-1 in mice results in spontaneous myocarditis and dilated cardiomyopathy driven by anti-cTn autoantibodies, while CTLA-4 knockout mice quickly die from autoimmune myocarditis mediated by CD8+ T cells. Additionally, myocardial PD-L1 overexpression has been observed in T-cell-mediated myocarditis mouse models. This upregulation is likely a cytokine-induced cardioprotective mechanism and may be reversed by an anti-PD-L1 antibody [6,10,11,12,13,14,15,16,17,18,19,20,21,22,23,24,25,26,27,28,29,30,31,32,33,34,35,36,37,38,39,40,41,42,48,49,50,51,52,53,54].

### 6.2. Management

The management of cardiac immune-related adverse events (irAEs) involves a multidisciplinary approach that includes close monitoring of cardiac function, early identification of symptoms, and prompt initiation of therapy. The first step in managing cardiac irAEs is to identify and grade the severity of the event. This is usually done using clinical examination, electrocardiogram (ECG), and cardiac biomarkers. The treatment of cardiac irAEs is mainly supportive and includes the use of corticosteroids, immunosuppressive agents, and/or IV immunoglobulin. In some cases, it may be necessary to temporarily interrupt or discontinue the immune checkpoint inhibitors (ICIs) therapy. Close monitoring of cardiac function is necessary during and after treatment, and includes frequent ECGs, echocardiograms, and biomarker evaluations.

Patients with moderate or severe cardiac irAEs may require hospitalization for close monitoring and management. In some cases, cardiology consultation and/or referral to a specialist center may be necessary. In addition, patient education on recognizing symptoms and the importance of timely follow-up is essential for early identification and management of cardiac irAEs [6,9,10,11,12,13,14,15,16,17,18,19,20,21,22,23,24,25,26,27].

The National Comprehensive Cancer Network (NCCN) guidelines also emphasize that the management of irAEs should be individualized and guided by the patient’s clinical presentation and cardiac function, and that the management of these events is still evolving. In addition, close communication and coordination between the oncologist and cardiologist is crucial for effective management of cardiac irAEs [35].

In the presented case, a 55-year-old female patient who was diagnosed with metastatic malignant melanoma in 2017 received a combination of immunotherapy with ipilimumab and nivolumab. However, the patient developed a grade 3 myocarditis. Myocarditis grading ranges from grade 1 to 4. The grades of severity are listed according to the to the American Society of Clinical Oncology clinical practice guidelines for the management of immune-related adverse events [38,45].

***Grade 1***—Abnormal cardiac biomarkers or electrocardiogram.
***Grade 2***—Mild symptoms with abnormal cardiac biomarkers and electrocardiogram.
***Grade 3***—TTE with LVEF < 50% or regional wall motion cardiac MRI diagnostic or suggestive of myocarditis.
***Grade 4***—Life-threatening disease with cardiac study abnormalities in grades 1–3 [35,48].

Current treatment guidelines for immune-related cardiac adverse events from ICIs differ between grades of toxicities. In the case of myocarditis grade 3, ICI should be suspended and high dose corticosteroids such as prednisone (1–2 mg/kd/d) initiated for 4–6 weeks [6,22,23,24,25]. Improvement should be seen within 3 days; if that does not occur, infliximab can be introduced regardless of heart failure status [6].

Following the development of ICI-related grade 3 myocarditis, we discontinued the immunotherapy and initiated corticosteroid therapy until full recovery. The patient was closely monitored and had regular cardiac MRI, and blood tests for troponin and pro-BNP.

In September 2018, a wide excision of the primary tumor was performed. Two months’ post-surgery, a follow-up PET–CT scan revealed no evidence of disease and the patient underwent regular monitoring by a cardiologist, oncologist, and immunologist, including monthly cardiac MRI and Echo as well as blood tests for troponin and pro-BNP.

Repeated treatment with ICI in patients with past ICI-related myocarditis is not recommended after stage 3 of myocarditis [6,34,36,38]. However, in April 2021, new metastases were observed in the patient’s upper and middle back and a biopsy confirmed malignant melanoma. A multi-disciplinary team comprising of an oncologist, cardiologist, and immunologist was convened to determine the appropriate course of treatment, taking into consideration the patient’s history of immune-related adverse effects and the NCCN guidelines, which recommend permanent discontinuation of immunotherapy in such cases and the initiation of alternative treatment options [35], but also keeping in mind that the guidelines also emphasizes that the management of immune-related adverse effects should be individualized and guided by the patient’s clinical presentation and cardiac function.

Given the patient’s history of complete response to the initial course of nivolumab and the availability of case reports and studies describing successful re-initiation of the same immunotherapy agent in patients with a complete response but discontinued therapy due to immune-related adverse events—for example, a retrospective, ‘real-world’ database study published in 2020, comprising mostly community oncology practices concluded that retreatment with anti-PD-1 monotherapy may provide additional benefit for patients with advanced melanoma that had responded to a first course of anti-PD-1 monotherapy with CR, PR, or SD [38,45]—it was decided to re-initiate monotherapy with nivolumab in order to minimize the risk of further adverse effects. The patient was closely monitored by a cardiologist, immunologist, and oncologist and as of December 2022, the patient had completed three cycles of therapy without reoccurring myocarditis and a recent (August 2023) PET–CT scan showed a complete response to treatment. In addition, it should be noted, that our patient has a medical history of diabetes mellitus and hypertension, which could potentially have a detrimental impact on heart function during Immune Checkpoint Inhibitor treatments. This medical history might be one of the contributing factors, potentially increasing the likelihood of our patient developing myocarditis [43,44,45,46,47,48,49,50,51,52,53,54,55,56,57,58].

Our patient had a high eosinophil count (eosinophilia) during diagnosis. Furthermore, it is worth noting that despite presenting with eosinophilia, the patient in the current article did not exhibit typical signs of Drug Reaction with Eosinophilia and Systemic Symptoms (DRESS) syndrome, such as rash and lymphadenopathy. Instead, it is important to consider the possibility of hypereosinophilic syndrome, also known as hypersensitivity (or allergic) myocarditis or idiopathic hypereosinophilic syndrome. In cases of DRESS syndrome, re-challenging the patient with the suspected medication is contraindicated because of the potentially life-threatening nature of the reaction. However, in the context of ICI myocarditis, it might be considered as an option for a selected group of patients, similar to the case reported. This is a rare condition characterized by clonal proliferative hematopathy originating from bone marrow progenitor cells. The DRESS-related myocarditis has similarly high mortality of around 40% [59,60,61,62,63]. It can be triggered by factors such as drug hypersensitivity, autoimmune diseases, idiopathic hypereosinophilic syndrome, or malignancy [59,60,61,62,63,64]. Additionally, it is crucial to highlight DRESS syndrome. The exact pathogenesis of DRESS syndrome remains poorly understood and is believed to involve complex interactions. Several drugs, including Lamotrigine, Allopurinol, Minocycline, and Abacavir, have been associated with DRESS syndrome. DRESS syndrome exhibits a wide array of clinical features. These clinical manifestations do not typically manifest immediately but usually become evident 2 to 8 weeks after the introduction of the triggering drug. Common features include fever, rash, lymphadenopathy, hematological abnormalities (such as eosinophilia and leukocytosis), and abnormal liver function tests. Managing DRESS syndrome hinges on prompt recognition and the withdrawal of the causative drug. It has been reported that earlier drug withdrawal leads to a more favorable prognosis. Treatment primarily involves providing support and alleviating symptoms; corticosteroids are often employed, although there is limited evidence regarding their efficacy. In some cases, additional immunosuppressants like cyclosporin may be necessary [59,60,61,62,63,64,65,66,67]. Myocarditis represents a relatively frequent yet often overlooked consequence of DRESS, which may manifest long after the initial diagnosis. In these instances, myocarditis can take on two distinct forms: hypersensitivity myocarditis (also recognized as acute eosinophilic myocarditis) and acute necrotizing eosinophilic myocarditis. Typically, hypersensitivity myocarditis demonstrates a self-limiting prognosis once the responsible agent is discontinued, and immunotherapy effectively suppresses the hypersensitivity reaction. The precise origins of DRESS-associated myocarditis remain elusive but are likely multifaceted, involving patient-specific factors, drug metabolites, and potential viral reactivation. One suggested cause is the buildup of harmful drug metabolites in individuals deficient in certain detoxifying enzymes. The diagnosis of hypersensitivity myocarditis is established through endomyocardial biopsy, revealing the presence of an eosinophilic and mixed lymphohistiocytic infiltrate, with no signs of necrosis or fibrosis [60,61,62,63,64,65,66].

It has been reported that myocarditis is an infrequent but serious complication of Influenza infection, characterized by a poor prognosis. The root cause of this poor prognosis often appears to be fulminant myocarditis, which can lead to sudden cardiac death [65,66]. In line with our approach, cases of Influenza-induced myocarditis are typically managed with corticosteroids, and in severe instances, intravenous immunoglobulin (IVIG) is administered. When comparing the incidence and mortality rates of myocarditis associated with immune checkpoint inhibitors (ICI) versus drug-induced myocarditis (DRESS) and infectious myocarditis (Influenza), the data reveal significant differences. Myocarditis linked to immune checkpoint inhibitors is relatively rare, with an incidence ranging from 0.04% to 1.14% [6]. However, it carries a significantly higher mortality rate, falling between 25% and 50% [36]. In contrast, clinical diagnoses of Influenza-related myocarditis have been reported in 5–10% of cases, with recent mortality rates ranging from 24% to 35% [36,38,39,40,41,42,43,44,45,46,47,48,49,50,51,52,53,54,55,56,57,58,59,60,61,62,63,64,65,66,67,68,69]. The most recent review suggests a reduced mortality rate of 14.7% [70]. Studies on DRESS historically indicate a relatively low incidence of cardiac involvement of around 13.3% [71]. Nevertheless, myocarditis associated with DRESS is associated with a similarly high mortality rate, approximately 40% [72]. When comparing the rarity of Immune Checkpoint Inhibitor-Induced Myocarditis to COVID-19-associated myocarditis, this discrepancy may stem from challenges related to misclassification and the intricate diagnosis of cardiac events triggered by ICIs, or other rare and unexpected adverse events related to the COVID-19 vaccine, particularly amid the ongoing COVID-19 pandemic [73,74,75,76,77,78]. A systematic review encompassing 43 articles, ultimately identified 51 patients with COVID-19-associated myocarditis based on clinical diagnosis. Notably, common clinical signs and symptoms included tachycardia, dyspnea, shock, and fever. Intriguingly, COVID-19-associated myocarditis demonstrated a significantly high mortality rate of 14.0% within the cases reviewed. In contrast, Immune Checkpoint Inhibitor-Induced Myocarditis is a rare occurrence, with an incidence of up to 1%. Nevertheless, it represents the most lethal form of cardiotoxicity, boasting a staggering mortality rate of up to 50%. Frequently reported clinical manifestations included shortness of breath and signs indicative of congestive heart failure, such as fatigue, edema, wheezing, weakness, and palpitations [6,36,79].

## 7. Conclusions

Immune checkpoint inhibitors can lead to various immune-related side effects, with one uncommon but severe occurrence being myocarditis. Although these side effects are infrequent, they carry a high risk of mortality. Hence, it is crucial to be vigilant about recognizing common symptoms and relevant laboratory results when treating patients. Furthermore, current protocols for monitoring, particularly in re-challenge situations, remain unclear. In this article, we demonstrated that re-challenge might be a viable option when coupled with diligent follow-up and comprehensive monitoring. This proactive approach ensures that patients can continue benefiting from the established anti-tumor effects of immune checkpoint inhibitors while effectively managing any adverse events that may arise.

## Data Availability

The data either resides within the article itself or can be obtained from the authors upon making a reasonable request.
